# Book Notices

**Published:** 1892-06

**Authors:** 


### Book Notices.

The Harvard Medical School Association has issued an interesting and valuable list to its members, which it will be glad to send to graduates of the Medical Department of Harvard University, in whatever part of the world they may be. The Association was formed about one year ago, and *all* graduates of the school are eligible to membership. The object is to unite all alumni and to advance the interests of the School of Medicine. The entrance fee and the annual assessment is merely nominal.

Bret Harte's young daughter, Miss Jessamy Harte, will make her literary debut in the July *Ladies' Home Journal* with a most entertaining description of "Camp Life in the Adirondacks," in which it is claimed every evidence shows itself of inherited literary tendencies not unlike those evidenced in Bret Harte's earlier work. Miss Harte is a girl still in her teens, and has artistic as well as literary proclivities, as one of the illustrations accompanying her first article shows.

Mr. Howells intends spending his summer in a quiet nook in New England, devoting a large portion of his time to the writing of his novel of American girl-life, to be published in the autumn in the *Ladies' Home Journal.*

THE POCKET MATERIA MEDICA AND THERAPEUTICS.—A resume of the Action and doses of all officinal and non-officinal drugs now in common use. By C. Henri Leonard, A. M., M. D., Detroit, Mich., 1891. Price $1.00.

INTERNATIONAL MEDICAL MAGAZINE has made its appearance, the initial number being dated February, 1892. It is a neatly printed octavo of 112 pages, edited by Dr. Judson Daland, and published by the Lippincott Company, of Philadelphia, monthly, at the price of $4 per year. It is a neatly gotten up journal, with excellent illustrations, and containing valuable matter.

